# 1684. Antimicrobial Activity of Aztreonam-Avibactam Against a Large Collection of *Stenotrophomonas maltophilia* and *Burkholderia cepacia* Species Complex Causing Infections in United States (US) Medical Centers (2016-2021)

**DOI:** 10.1093/ofid/ofac492.1314

**Published:** 2022-12-15

**Authors:** Helio S Sader, Dee Shortridge, S J Ryan Arends, Cecilia G Carvalhaes, Rodrigo E Mendes, Mariana Castanheira

**Affiliations:** JMI Laboratories, North Liberty, Iowa; JMI Laboratories, North Liberty, Iowa; JMI Laboratories, North Liberty, Iowa; JMI Laboratories, North Liberty, Iowa; JMI Laboratories, North Liberty, Iowa; JMI Laboratories, North Liberty, Iowa

## Abstract

**Background:**

*S. maltophilia* has become a major cause of hospital-associated pneumonia. Aztreonam (ATM) is a monobactam stable to hydrolysis by metallo-β-lactamases (MBLs), including those intrinsically produced by *S. maltophilia*. Avibactam (AVI) is a non-β-lactam β-lactamase inhibitor that inhibits serine β-lactamases such as ESBLs, KPCs, AmpCs, and some OXAs. ATM-AVI is being developed for treatment of serious infections caused by Gram-negative bacteria, including MBL producers. We evaluated the activity of ATM-AVI against *S. maltophilia* and *B. cepacia* from US hospitals.

**Figure:**
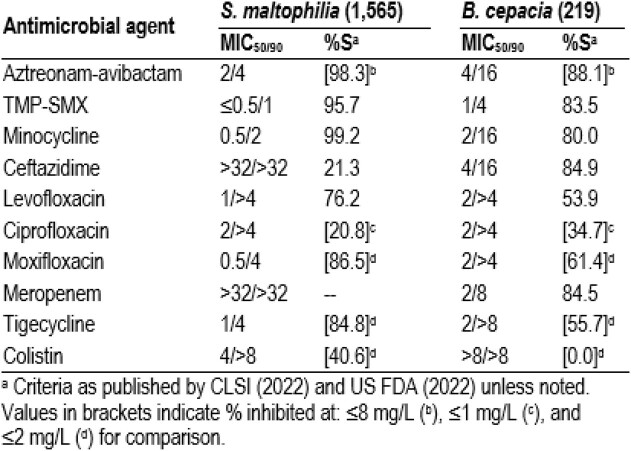

**Methods:**

1,565 *S. maltophilia* and 219 *B. cepacia* were consecutively collected (1/patient) in 77 US medical centers in 2016-2021 and susceptibility tested by CLSI broth microdilution method. Only isolates determined to be the probable cause of infection were included. CLSI/US FDA breakpoints were applied when available.

**Results:**

The isolate collection were recovered mainly from patients with pneumonia (73.3%) and bloodstream infection (BSI; 10.1%). ATM-AVI was very active against *S. maltophilia*, with MIC_50/90_s of 2/4 mg/L and 98.3% of isolates inhibited at ≤8 mg/L, including 100.0% of isolates from BSI and 97.9% of isolates from pneumonia. Trimethoprim-sulfamethoxazole (TMP-SMX; MIC_50/90_, ≤0.5/1 mg/L; 95.7% susceptible [S]) and minocycline (MIC_50/90_, 0.5/2 mg/L; 99.2%S) also were very active against *S. maltophilia*, while ceftazidime and levofloxacin were active against 21.3% and 76.2% of isolates per CLSI criteria, respectively (Table). ATM-AVI was also very active against *B. cepacia*, with MIC_50/90_s of 4/16 mg/L and 88.1% inhibited at ≤8 mg/L, including 93.3% and 87.3% of isolates from BSI and pneumonia, respectively. The most active comparators tested against *B. cepacia* were ceftazidime (84.9%S), meropenem (84.5%S), TMP-SMX (83.5%S), and minocycline (80.0%S).

**Conclusion:**

ATM-AVI demonstrated potent *in vitro* activity against *S. maltophilia* and *B. cepacia* from US hospitals and may represent a valuable option to treat infections caused by these organisms. Clinical studies are urgently warranted to evaluate the efficacy of ATM-AVI as well as reevaluate the susceptibility breakpoints for antibiotics currently used to treat infections caused by these organisms.

**Disclosures:**

**Helio S. Sader, MD, PhD**, AbbVie: Grant/Research Support|Cidara: Grant/Research Support|Melinta: Grant/Research Support|Nabriva Therapeutics: Grant/Research Support|Pfizer: Grant/Research Support **Dee Shortridge, PhD**, AbbVie: Grant/Research Support|JMI Laboratory: Employee|Melinta: Grant/Research Support|Menarini: Grant/Research Support|Shionogi: Grant/Research Support **SJ Ryan Arends, PhD**, AbbVie: Grant/Research Support|GSK: Grant/Research Support|Nabriva Therapeutics: Grant/Research Support|Shionogi: Grant/Research Support **Cecilia G. Carvalhaes, MD, PhD**, AbbVie: Grant/Research Support|Cidara: Grant/Research Support|Melinta: Grant/Research Support|Pfizer: Grant/Research Support **Rodrigo E. Mendes, PhD**, AbbVie: Grant/Research Support|Cidara: Grant/Research Support|GSK: Grant/Research Support|Melinta: Grant/Research Support|Nabriva Therapeutics: Grant/Research Support|Office for Assistant Secretary of Defense for Health Affairs: Grant/Research Support|Pfizer: Grant/Research Support|Shionogi: Grant/Research Support|Spero Therapeutics: Grant/Research Support **Mariana Castanheira, PhD**, AbbVie: Grant/Research Support|Cidara: Grant/Research Support|GSK: Grant/Research Support|Melinta: Grant/Research Support|Pfizer: Grant/Research Support|Shionogi: Grant/Research Support.

